# Why do apprentices smoke much more than high school students? Understanding educational disparities in smoking with a Oaxaca-blinder decomposition analysis

**DOI:** 10.1186/s12889-020-09050-4

**Published:** 2020-06-12

**Authors:** Sandra Chyderiotis, Tarik Benmarhnia, Stanislas Spilka, François Beck, Raphaël Andler, Stéphane Legleye, Gwenn Menvielle

**Affiliations:** 1 Université Paris-Sud, Université Paris-Saclay, faculté de médecine, faculté de médecine UVSQ, Inserm, CESP, 92541 Villejuif, France; 2 Observatoire français des drogues et des toxicomanies (OFDT), 69, rue de Varenne, 75007 Paris, France; 3 Department of Family Medicine and Public Health, University of California, San Diego, 9500 Gilman Drive, La Jolla, CA 92093 USA; 4 Scripps Institution of Oceanography, University of California, San Diego, CA USA; 5 Institut national de la statistique et des études économiques, 88, avenue Verdier, CS 70058, 92541 Montrouge Cedex, France; 6 Santé Publique France, 12, rue du Val d’Osne, 94415 Saint-Maurice Cedex, France; 7 Department of Social Epidemiology, Sorbonne Université, INSERM, Institut Pierre Louis d’Epidémiologie et de Santé Publique IPLESP, 75012 Paris, France

**Keywords:** Adolescence, Education, Apprenticeship, Health disparities, Smoking, Decomposition analysis, Oaxaca-blinder, France

## Abstract

**Background:**

Educational disparities in daily smoking begin during adolescence and can lead to educational disparities in health among adults. In particular, vocational students including apprentices have higher daily smoking rates compared to non-vocational students. This study aimed to identify the determinants of the gap in daily smoking between French apprentices and high school students aged 17 in 2008 and in 2017.

**Methods:**

We used data from a cross-sectional repeated survey representative of all French adolescents aged 17 in 2008 and 2017. We conducted a non-linear extension of the Oaxaca-Blinder decomposition technique and included the following variables: sociodemographic and familial characteristics, parental smoking, cannabis and alcohol use, suicidal attempt, grade repetition and money received.

**Results:**

Daily smoking was about two times higher among French apprentices compared to high school students in 2008. This gap did not decrease between 2008 and 2017. Differences in measured characteristics between the two groups explained this gap partly, from 28.6 to 51.2%. Cannabis and alcohol use, money received and parental smoking contributed the most to the daily smoking gap.

**Conclusions:**

Prevention programs could target cannabis and alcohol use as well as parental smoking to help decrease educational disparities in smoking status among French adolescents.

## Background

Despite encouraging decreases in smoking prevalence, educational disparities in smoking have increased in the past decades in many Western countries [1], notably in France [2, 3]. This increase reflects a drop in smoking prevalence concentrated among people with the highest levels of education [4, 5]. Educational disparities in smoking is a major public health concern as smoking is among the largest contributors to educational disparities in morbidity and mortality in Western countries [6].

Educational disparities in daily smoking are already present in adolescence [7], depicting an earlier initiation among those with lower education. In particular, students with vocational training have been shown to have higher prevalence rates of tobacco smoking than non-vocational students in various Western countries [8–12]. This could result from differences between students in characteristics such as family socioeconomic status, personal and family difficulties [7]. Alternatively, this could arise from some specificities of vocational training such as a closer proximity with the adult-type lifestyles and the professional environment, often in manual employment where, in France, smoking rates are high [3, 13], and higher financial resources [14].

Disentangling and quantifying the relative importance of each of those characteristics on educational disparities in smoking among adolescents can provide insights to design tailored interventions aimed at reducing these disparities. The Oaxaca-Blinder decomposition method [15, 16] is a statistical tool adapted for this purpose, and has commonly been used to study sources of disparities in health, including smoking behaviours [17–19] and adolescent health [20, 21], but has not yet been applied to educational disparities in smoking among adolescents. The Oaxaca-Blinder decomposition method decomposes a given disparity between 2 groups into a portion due to a statistical variation in the covariates of interest (the explained portion) and an unexplained portion (that includes the effect of unmeasured variables) [22]. The explained portion corresponds to potential changes in the disparity of interest when equalizing identified measured covariates of interest. Said differently, such decomposition technique quantifies the potential reduction in the disparity for a given outcome between two groups under a hypothetical scenario where both groups would have the same value for each covariate of interest.

In France, many tobacco control policies have been implemented since 2008 [23]. Some policies were specifically targeting youths such as a ban on the sale to minors (2009). Others applied to the whole population including visual health messages (2011) and regular price increases. These policies and societal changes could have modified the educational gap in daily smoking among adolescents as well as the variables accounting for this gap. Indeed, tobacco control policies have been shown to affect health inequalities although more research is needed in this field [24, 25]. It is thus critical to assess changes in educational disparities in smoking in French adolescents.

To better understand educational disparities in smoking among adolescents, we took advantage of a unique, nationally representative, French survey that includes adolescents aged 17. We employed a nonlinear Oaxaca Blinder decomposition approach to quantify how much of the difference in daily smoking rates between high school students and apprentices could be attributed to differences in observed characteristics between the two groups or to the apprenticeship setting. We compared data from 2008 and 2017 to investigate potential evolutions over the past 10 years.

## Methods

### Data

The ESCAPAD survey (*Enquête sur la Santé et les Consommations lors de l’Appel de Préparation A la Défense*) is a repeated cross-sectional, standardized, nationally representative survey that provides estimates of prevalence of drug use among French adolescents aged 17 [26].

The ESCAPAD survey is conducted by the French Monitoring Centre for Drugs and Drug Addiction and the Department of National Civil Service and Youth during the National Defence and Citizenship Day (JDC): a one-day session of civic and military information that is compulsory for later enrolment in public exams (i.e. driver’s license, university exams, etc.). All French nationals are summoned to participate shortly after having turned 17. About 4% of youths never attend the JDC.

The ESCAPAD survey takes place during 2 weeks in March in all centres across the French territory where the JDC is organized. All of the adolescents present are invited to participate in the survey. The pen and paper questionnaires are self-administered, anonymous and follow the recommendations of the European Monitoring Centre for Drugs and Drug Addiction [27]. Participants are guaranteed complete confidentiality and anonymity and can refuse to complete the form. The survey was approved by the National Council for Statistical Information (CNIS), as well as the ethics commission of the French National Data Protection Authority (CNIL).

Our analysis focuses on the 2008 and the 2017 waves of the ESCAPAD survey. Response rates were consistently above 90% (number of filled questionnaires out of number of adolescents who attended the JDC the day of the survey). Respondents aged 17 consistently represented more than 90% of the samples, the others were aged 18. The data are calibrated to guarantee the weight of 17 years-olds in each “département” (official geographic and administrative unit) and their sex ratio inside each “département”.

### Study population

We compared two educational tracks: high school (starting after middle school, around age 15) and apprenticeship. In France, apprenticeship contracts are work contracts opened to people aged 16 to 29. They combine 2 weeks of in-company training alternated with 1 week of courses [28]. Apprentices are at the same time students and employees, often in manual employment, and thus have regular contacts with an older and professional environment and receive a salary. Most apprentices enter the workforce after their diplomas, while most high school students enter tertiary education.

### Measures

Our outcome variable was daily smoking at the time of the survey. This was measured through the question “during the last 30 days, have you smoked cigarettes?” with the following choices: never; less than once a week; less than once a day; 1–5 per day; 6–10 per day; 11–20 per day; more than 20 per day. People who declared smoking one or more cigarettes per day in the past 30 days were considered daily smokers.

The independent variables included the following demographic and familial characteristics: gender (girl/boy), age (as a continuous variable), living with no relatives (yes/no), parents living together (yes/no), father and mother smoking status (occasional or current smoker yes/no) and parental occupational status. Parental occupational status was defined as the highest occupational category of the parents, as reported by adolescents, based on the National Institute for Statistics and Economic Studies’ official typology (more details can be found elsewhere [29]). This yielded a final 5-category scale: low (both parents are unemployed or inactive, or both situations are missing), disadvantaged (the highest parental occupational category is manual worker or clerk), intermediate (highest parental occupational category is intermediate: technician and associate professional), advantaged (one parent is a manager, or has a liberal or intellectual occupation), high (parents are both managers, or have a liberal or intellectual occupation). In addition, suicidal attempts that lead to hospitalization in the lifetime (yes/no) were included as a proxy measure of mental health. We approached school performance by the question “Have you ever repeated a class?” (No; yes). We accounted for the use of cannabis (yes/no) and use of alcohol (yes/no) in the past month and the amount of money received in the past month (as a continuous variable). In 2008, this amount was obtained through three subquestions distinguishing pocket money, salary from a job (baby-sitting etc.) or from an internship or apprenticeship, and money for a special occasion. In 2017, salary from a job (baby-sitting etc.) was additionally distinguished from salary received from an internship or apprenticeship. The amount received superior to zero was capped at the 99th percentile for each subquestion to avoid obvious irrelevant reports. In 2017, parental smoking was available only for a subsample (thereafter called 2017-A) and the amount of money received only for another subsample (thereafter called 2017-B). Age at smoking initiation (“What was your age the first time you smoked a cigarette”?) and age at transition to daily smoking (“If you are smoking daily, at what age did you start smoking every day?”) were also measured but not included in the Oaxaca-Blinder models due to the correlation with our outcome.

In 2008, the survey included 39,542 adolescents (33,253 high school students, 4564 apprentices, 1725 school dropouts and 0 with missing education). In 2017, the subsample 2017-A included 12,471 adolescents (11,093 high school students, 877 apprentices, 459 school dropouts and 42 with missing education) and the subsample 2017-B 13,314 adolescents (11,769 high school students, 949 apprentices, 546 school dropouts and 50 with missing education). Those who dropped out of school and those who did not fill their educational background were excluded from the analyses.

### Statistical analyses

First, we compared the distribution of all independent variables between apprentices and high school students as well as the daily smoking rate for each characteristic separately for apprentices and high school students. To do so, we calculated the absolute standardized mean differences (SMD) since they are not influenced by sample size [30].

Then, we applied a nonlinear Oaxaca-Blinder decomposition, designed for nonlinear outcomes. This approach decomposes the observed difference in daily smoking between apprentices and high school students into an explained and an unexplained part [31]. High school students were used as the reference group. The explained part is the part of the daily smoking gap associated with differences in measured characteristics between two groups. It shows the expected change in daily smoking gap that would be observed if apprentices were given the covariates’ distribution of high school students. The contribution of each variable to the daily smoking gap was reported as log odds and percentage contribution (percentage of the total difference). The explained part expressed in % is the proportion of the total disparity that would be reduced following a hypothetical intervention equalizing the average value of each covariate in both groups. The explained part thus quantifies the change in daily smoking disparities that would be observed if the averaged values for each covariate of interest were set to be equal between apprenticeships and high school students.

The unexplained part depicts, in addition to the effect of unmeasured characteristics, the heterogeneity across educational groups in the effect of characteristics on the smoking outcome, as well as the effect of the apprenticeship environment on educational disparities in smoking.

All analyses were performed with Stata 15. To take into account the “identification problem” that arises when including categorical variables in the model [32], we used the -oaxaca- command in Stata with the options ‘logit’, ‘weight(0)’ and ‘normalize’ [33]. We compared with results obtained using the command -mvdcmp- with the option ‘normal’ [34] and did not find any difference.

The decomposition analysis was conducted for the 2008 survey, the 2017-A and the 2017-B subsamples separately. Because of concurrent use of cannabis and alcohol use with daily smoking in the last 30 days, we performed sensitivity analyses by removing these two variables. In addition, for comparison purposes, we performed two decomposition analyses using the 2008 survey, each including similar variables as the one in the subsamples 2017-A and 2017-B (See Tables 1S and 2S, Additional File 1). Finally, we tested the robustness of our models by decomposing educational disparities in daily smoking for each independent characteristic separately (See Table 3S, Additional File 1).

## Results

### Descriptive statistics

Table 1 displays descriptive statistics of apprentices and high-school students aged 17. Regardless of the sample, compared to high school students, apprentices have received much more money, are more likely to be boys, to have repeated a class, to have used cannabis and alcohol in the past month, as well as to have parents who smoke, have a low occupational status, and do not live together. In the subsample 2017-B, apprentices were more likely to live alone than high school students. Suicide attempts were more frequent among apprentices in 2008 and in the subsample 2017-B. Daily smoking rates were about two times higher among apprentices than among high school students: 49.9% compared to 24.5% in 2008, 44.8% compared to 22.1% in the subsample 2017-A and 46.8% compared to 21.9% in the subsample 2017-B. Age at smoking initiation and especially age at transition to daily smoking were earlier for apprentices of all samples.

**Table 1:** Characteristics of high school students and apprentices in the 2008, 2017-A and 2017-B samples. ESCAPAD survey, OFDT

	2008 sample			2017-A sample			2017-B sample		
	High school students (*n* = 33,253)	Apprentices (*n* = 4,564)	SMD	High school students (*n* = 11,093)	Apprentices (*n* = 877)	SMD	High school students (*n* = 11,769)	Apprentices (*n* = 949)	SMD
Age (mean)	17.3	17.4	0.17	17.4	17.4	0.04	17.4	17.4	0.02
Daily smoking (Yes)	24.5	49.9	0.54	22.1	44.8	0.50	21.9	46.8	0.54
Age at smoking initiation (mean)	13.7	13.2	0.20	14.6	14.0	0.30	14.5	13.9	0.32
Age at transition to daily smoking (mean)	15.0	14.5	0.34	15.3	14.9	0.35	15.2	14.7	0.41
Gender (Boys)	47.9	70.2	0.47	49.0	73.7	0.52	49.4	73.8	0.52
Parental occupational status			0.41			0.47			0.46
Low	10.3	16.2	0.17	9.6	14.5	0.15	9.8	15.2	0.16
Disadvantaged	31.0	44.0	0.27	34.6	46.0	0.23	34.6	46.5	0.25
Intermediate	16.2	14.2	0.05	23.8	26.2	0.06	23.8	25	0.03
Advantaged	29.6	20.3	0.22	21.6	11.9	0.26	21.5	10.6	0.30
High	12.8	5.3	0.26	10.5	1.4	0.39	10.4	2.6	0.32
Parents living together (Yes)	72.0	62.7	0.20	64.6	54.9	0.20	64.8	54.5	0.21
Living alone (Yes)	9.9	10.9	0.03	8.3	9.9	0.06	8.0	12.4	0.15
Suicide attempt (Yes)	1.7	3.2	0.10	2.5	2.6	0.01	2.5	4.1	0.09
Grade repetition (Yes)	39.1	70.3	0.66	26.3	53.1	0.57	26.6	55.8	0.62
Cannabis use in the past month (Yes)	23.1	32.7	0.22	19.7	28.7	0.21	19.6	28.8	0.22
Alcohol use in the past month (Yes)	76.9	83.3	0.16	66.0	75.4	0.21	65.6	78.7	0.29
Father smokes (Yes)	35.6	46.6	0.22				32.6	45.3	0.26
Mother smokes (Yes)	30.4	42.4	0.25				29.5	43.4	0.29
Sum of money received^1^			1.60			1.74			
Quintile 1	23.7	4.6	0.57	25.8	2.5	0.71			
Quintile 2	21.6	3.1	0.58	19.1	1.9	0.58			
Quintile 3	23.3	3.0	0.63	19.6	2.1	0.58			
Quintile 4	21.0	5.9	0.45	21.3	3.0	0.58			
Quintile 5	10.3	83.3	2.14	14.3	90.5	2.36			

*SMD* Absolute standardized mean difference

1: The quintiles were computed for each survey among the whole population. For 2008, sum of money was distributed into quintiles (1: [0–15€], 2: [16-30], 3: [31–70], 4: [71–200€], 5: [201 and more]. For 2017, the distribution was (1: [0–20€], 2: [22-50], 3: [51–120], 4: [121–300€], 5: [302 and more]

Table 2 presents daily smoking rates at 17 for each characteristic separately for apprentices and high school students and thus showcases the differences in the covariates of interest between apprenticeships and high school students. The same smoking profile was observed for high school students and apprentices for most variables: higher daily smoking rates were reported among adolescents whose parents smoked or did not live together as well as among adolescents who lived alone, reported at least one suicidal attempt in their lifetime, and used cannabis or alcohol in the past month. Daily smoking rates also gradually increased with the amount of money received in both groups. Repeating a class was associated with higher daily smoking rates among high school students but not among apprentices. Both among high school students and apprentices, parental occupational status did not clearly affect daily smoking rates. In the subsample 2017-A, female apprentices smoked less than male apprentices (38.1% vs 47.3%).

**Table 2:** Daily smoking rates among high school students and apprentices according to various characteristics for the 2008, 2017-A and 2017-B samples. ESCAPAD survey, OFDT

	2008 sample				2017-A sample				2017-B sample			
	High school students		Apprentices		High school students		Apprentices		High school students		Apprentices	
	Daily smoking	SMD	Daily smoking	SMD	Daily smoking	SMD	Daily smoking	SMD	Daily smoking	SMD	Daily smoking	SMD
Gender		0.05		0.01		0.05		0.13		0.04		0.08
Boys	23.7		50.6		21.7		47.3		21.7		47.3	
Girls	25.3		48.3		22.44		38.1		22.2		45.4	
Parental occupational status		0.04		0.04		0.00		0.06		0.01		0.14
Low	26.6	0.05	52.9	0.01	22.5	0.00	43.8	0.00	22.6	0.02	43.5	0.12
Disadvantaged	25.6	0.03	48.9	0.02	22.1	0.00	42.2	0.05	21.7	0.02	46.2	0.04
Intermediate	22.8	0.05	46.2	0.04	21.8	0.03	47.2	0.01	22.5	0.04	45.8	0.06
Advantaged	23.8	0.01	51.6	0.02	23.3	0.06	50.0	0.07	22.6	0.03	54.7	0.06
High	24.1	0.00	52.7	0.06	19.8	0.04	54.3	0.03	19.1	0.03	52.8	0.05
Parents living together		0.25		0.21		0.25		0.27		0.27		0.15
No	32.3		57.7		28.4		52.1		28.5		51.7	
Yes	21.5		45		18.6		38.6		18.3		42.4	
Living alone		0.18		0.19		0.18		0.12		0.12		0.10
No	23.2		48		21.0		44.0		21.1		45.6	
Yes	36.0		64.8		33.7		54.7		30.2		53.2	
Suicide attempt		0.24		0.27		0.21		0.13		0.23		0.13
No	23.4		47.4		21.4		44.6		21.1		45.8	
Yes	65.2		85.5		47.5		54.5		53.7		68.4	
Grade repetition		0.37		0.10		0.26		0.03		0.25		0.00
No	19.0		45.9		19.2		44.4		19.2		46.9	
Yes	33.2		51.5		30.3		45.2		29.3		46.9	
Cannabis use in the past month		1.15		1.13		1.10		1.07		1.17		0.89
No	12.9		32.2		12.2		27.7		11.6		32.5	
Yes	62.4		84.5		61.2		84.3		64.0		80.2	
Alcohol use in the past month		0.47		0.42		0.67		0.42		0.68		0.45
No	10.2		26.5		7.6		25.6		7.8		25.4	
Yes	28.8		54.6		29.5		51.1		29.1		52.1	
Father smokes		0.33		0.30						0.35		0.31
No	19.4		42.2						16.7		36.4	
Yes	32.4		57.7						29.4		51.8	
Mother smokes		0.37		0.37						0.44		0.38
No	19.7		42.2						16.0		35.7	
Yes	35.3		60.3						32.4		55.0	
Sum of money received^1^		0.32		0.13		0.34		0.17				
Quintile 1	16.8	0.26	34.2	0.12	13.6	0.27	19.9	0.13				
Quintile 2	21.6	0.08	37.9	0.08	19.9	0.03	26	0.10				
Quintile 3	26.5	0.06	47.3	0.03	20.1	0.02	37.8	0.03				
Quintile 4	29.6	0.13	50.3	0.00	23.6	0.09	39.5	0.04				
Quintile 5	35.1	0.18	51.5	0.09	32.2	0.26	43.4	0.15				

*SMD* Absolute standardized mean difference

1: The quintiles were computed for each survey among the whole population. For 2008, sum of money was distributed into quintiles (1: [0–15€], 2: [16-30], 3: [31–70], 4: [71–200€], 5: [201 and more]. For 2017, the distribution was (1: [0–20€], 2: [22-50], 3: [51–120], 4: [121–300€], 5: [302 and more]

How to read: in 2008 among high school students, daily smoking rate was 25.3% among girls

### Oaxaca blinder decomposition analyses

Table 3 describes the results from the non-linear Oaxaca-Blinder decomposition analyses. The explained part varied from 28.6 to 51.2% depending on the sample and the model. This represents the share of the observed daily smoking gap between high school students and apprentices that could be attributed to differences in measured characteristics. The variables that accounted the most for the smoking gap between high school students and apprentices were, in decreasing order of importance, cannabis use in the past month (12.9 to 16.9%), sum of money received (8.7 to 12.8%), alcohol use in the past month (4.3 to 8.0%), mother’s smoking (4.4 to 5.2%) and father’s smoking (3.4 to 4.9%). In 2008, gender and suicide attempt also explained a small part of the smoking gap between high school students and apprentices. Parental occupational status, grade repetition and living alone consistently showed no contribution to educational disparities in smoking.

**Table 3:** Oaxaca-Blinder decomposition of differences in daily smoking between high school students and apprentices in the 2008, 2017-A and 2017-B samples: estimates and percentage contributions. ESCAPAD survey, OFDT

	2008 sample			2017-A sample			2017-B sample		
	Estimate	Share	CI	Estimate	Share	CI	Estimate	Share	CI
	Log odds	(%)		Log odds	(%)		Log odds	(%)	
Total Unexplained	−0.14	58.7	57.6 to 60.5	− 0.11	48.8	44.3 to 50.3	−0.15	71.4	66.6 to 83
Total Explained	−0.10	41.3	39.5 to 42.4	−0.11	51.2	49.7 to 55.7	−0.06	28.6	17 to 33.4
Age	0.00	1.6	0.4 to 2.3	0.00	0.0	−0.7 to 0.2	0.00	1.1	−1.3 to 2.1
Gender (Girl)	0.01	−3.7	−0.3 to −9.1	− 0.01	3.6	− 10.4 to 8.5	0.00	− 0.2	6.5 to 15.6
Parental occupational status									
Low	0.00	0.1	−0.8 to 0.7	0.00	0.9	−2.8 to 2.2	0.00	−0.5	−3.8 to 0.9
Disadvantaged	0.00	− 0.5	−2.9 to 1.1	0.00	2.2	−3.8 to 4.3	0.00	0.2	−7.2 to 3.3
Intermediate	0.00	0.1	−0.4 to 0.4	0.00	0.2	−1.7 to 0.9	0.00	0.2	−0.9 to 0.7
Advantaged	0.00	0.7	−0.8 to 1.6	0.00	−1.3	− 12 to 2.5	0.00	0.7	−8.1 to 4.4
High	0.00	−0.8	−3.6 to 1.0	− 0.01	5.6	−7.5 to 10.2	0.00	−0.4	−11.5 to 4.3
Parents living together (Yes)	0.00	0.4	−1.1 to 1.3	−0.01	2.6	−2.6 to 4.4	0.00	−0.2	−5.6 to 2.0
Living alone (Yes)	0.00	0.3	−0.2 to 0.7	0.00	0.9	−1.3 to 1.7	0.00	−0.4	−4.1 to 1.2
Suicide attempt (Yes)	0.00	1.7	0.7 to 2.3	0.00	−0.3	−3.5 to 0.8	0.00	1.1	−1.3 to 2.0
Grade repetition (Yes)	−0.01	4.2	−0.7 to 7.2	−0.01	2.6	−12.4 to 7.8	0.01	−4.0	−23.9 to 4.4
Cannabis in the past month (Yes)	−0.04	16.4	16.1 to 16.9	−0.04	16.9	16.8 to 17.2	−0.03	12.9	10.5 to 13.9
Alcohol in the past month (Yes)	−0.01	4.3	3.6 to 4.7	−0.01	4.5	1.1 to 5.6	−0.02	8.0	4.1 to 9.6
Father smokes (Yes)	−0.01	3.4	2.2 to 4.2				−0.01	4.9	−0.3 to 7.1
Mother smokes (Yes)	−0.01	4.4	3.3 to 5.0				−0.01	5.2	0.5 to 7.2
Sum of money received (continuous)	−0.02	8.7	−1.1 to 14.8	−0.03	12.8	−7.7 to 20.0			66.6 to 83.0

*CI* confidence interval

In the sensitivity analyses excluding alcohol and cannabis use (Table 4), the explained part decreased in each model. The share of the explained proportion increased for money received, gender, parents living together and parental smoking.

**Table 4:** Oaxaca-Blinder decomposition of differences in daily smoking between high school students and apprentices in the 2008, 2017-A and 2017-B samples: raw estimates and percentage contributions while excluding use of alcohol and cannabis. ESCAPAD survey, OFDT

	2008 sample			2017-A sample			2017-B sample		
	Estimate	Share	CI	Estimate	Share	CI	Estimate	Share	CI
	Log odds	(%)		Log odds	(%)		Log odds	(%)	
Total Unexplained	−0.16	65.2	62.8 to 69.4	−0.16	71.3	64.4 to 99.7	−0.18	82.1	74.8 to 99.7
Total Explained	−0.08	34.8	30.6 to 37.2	−0.06	28.7	0.3 to 35.6	−0.04	17.9	0.3 to 25.2
Age	−0.01	2.3	1.3 to 2.9	0.00	−0.1	−2.1 to 0.3	0.00	0.8	−1.3 to 1.7
Gender (Girl)	−0.01	2.3	−1.6 to 4.7	−0.02	10.9	3.4 to 12.7	−0.02	8.5	−1.4 to 12.6
Parental occupational status									
Low	0.00	−0.1	−1.2 to 0.5	0.00	−0.5	−7.6 to 1.2	0.00	−1.3	−5.7 to 0.5
Disadvantaged	0.00	−1.0	−3.8 to 0.7	0.00	−1.6	−15.2 to 1.7	0.00	0.2	−7.5 to 3.3
Intermediate	0.00	0.2	−0.3 to 0.5	0.00	−0.3	−3.9 to 0.6	0.00	0.2	−0.9 to 0.7
Advantaged	0.00	0.2	−1.6 to 1.2	0.00	−1.0	− 15.4 to 2.5	0.00	−0.5	− 10.4 to 3.6
High	0.00	−1.7	−5.2 to 0.3	0.01	−2.4	−32.1 to 4.9	0.00	−1.1	−12 to 3.4
Parents living together (Yes)	0.00	1.8	0.5 to 2.5	−0.01	6.1	4.6 to 6.5	0.00	0.2	−4.8 to 2.2
Living alone (Yes)	0.00	0.5	−0.2 to 0.9	0.00	0.9	−1.9 to 1.6	0.00	0.3	−2.9 to 1.6
Suicide attempt (Yes)	−0.01	2.5	1.4 to 3.1	0.00	−0.4	−5 to 0.8	0.00	1.3	−0.8 to 2.1
Grade repetition (Yes)	−0.01	2.5	−3.3 to 5.9	0.00	0.2	−23.4 to 6.0	0.01	−5.2	−26.3 to 3.5
Father smokes (Yes)	−0.01	4.1	3.1 to 4.7				−0.01	6.2	1.6 to 8.1
Mother smokes (Yes)	−0.02	6.3	5.8 to 6.6				−0.02	8.5	5.3 to 9.8
Sum of money received (continuous)	−0.04	15.0	6.0 to 20.3	−0.04	16.9	−11.1 to 23.7			74.8 to 99.7

*CI* confidence interval

The additional analyses for the 2008 survey using the same variables in 2008 as in the 2017 subsamples yielded similar findings (Tables 1S and 2S, Additional File 1). The explained share and the percentage contribution of the variables were quite similar in 2008 and in 2017, except for differences in 2008 already identified in the main analyses. Results from the additional analysis with each characteristic tested separately yielded consistent results (Table 3S, Additional File 1).

## Discussion

We found large educational differences in daily smoking among adolescents aged 17 in France, with daily smoking rates about two times higher in apprentices than in high school students. This gap hardly changed between 2008 and 2017.

We found that between 28.6 and 51.2% of the observed difference in daily smoking between high school students and apprentices could be attributed to differences in their characteristics. Overall, the explained share of each characteristic is relatively stable over the 10-year studied period. This is consistent with the unchanged smoking gap, but those results should be emphasized in relation to the tobacco control policies implemented during this period. This absence of change could be explained by two factors: first, tobacco control policies targeting adolescents (tobacco sales ban to minors, plain packaging) are recent in France [35] and it might take time before some can have an impact on smoking behaviours among adolescents. Second, none of the policies launched before the mid 2010’s were aimed at specifically reducing disparities in adolescent smoking. It may also be due to the fact that adolescents react similarly to smoking bans and price increases whatever their social background.

We found that the familial environment in which adolescents live could influence their smoking behaviours. Differences in parental occupational status between high school students and apprentices did not directly contribute to educational disparities in daily smoking for these two groups. On the contrary, differences in parental smoking between both educational groups, and to a lesser extent, familial difficulties associated with parents not living together (e.g. less stability in the household, less parental control, more conflicts or violence), seemed to be of importance in explaining the smoking gap. Those results are partly consistent with the current literature. Indeed, Alves et al. suggested that adolescents were more likely to smoke if their parents smoked and this association was similar across social classes [36]. However, contrary to our findings, parental socioeconomic class has been found to be an important determinant of smoking disparities in French youth as a whole [37]. This is nevertheless not always true in other contexts [38]. More research is needed to better understand the relationship between parental occupational status, parental smoking and adolescent smoking. Our results therefore suggest that interventions aimed at reducing smoking inequalities among parents [25, 39] as well as reinforcing life skills among adolescents [40], in order to help them deal with familial difficulties, could help reduce the smoking gap between high school students and apprentices.

Differences in money received accounted for a substantial part of the gap in daily smoking between high school students and apprentices. Disposable income would be a more informative variable, but was not available. Nevertheless, the amount of money received could reflect the higher financial autonomy of apprentices and their increased possibility to buy cigarettes. Indeed, as shown is Perelman et al., personal income is closely associated to smoking among adolescents [41]. However, apprentices receive much more money than high school students: about 83–90% of apprentices are in the highest quintile of money compared to only 10 to 14% of high school students. Therefore, we cannot exclude a specific effect of apprenticeship, and money is likely to be a marker for being an apprentice, including - but not limited to - financial autonomy.

The highest share of the explained part of educational differences in daily smoking was due to cannabis use in the last month, closely followed by alcohol use. As these behaviours are closely linked with daily smoking [42], it is understandable that if smoking is more prevalent in one group, use of alcohol and cannabis will also be. We thus conducted sensitivity analyses removing cannabis and alcohol use, which found lower overall explained parts. This can reflect the higher association of those three behaviours in the past month of the survey among apprentices compared to high school student, and maybe different ways of consuming tobacco, alcohol and cannabis in those two groups (types of products, frequency, link with social activities etc.). Qualitative studies focusing on those questions in those understudied populations could help better understand our results.

The contribution of some variables to the explained part nevertheless increased, such as gender and money received. This might highlight a specific relationship between those variables, smoking and cannabis and alcohol use. Finally, as those three behaviours are often associated, interventions focusing on addiction and psychoactive products in general could be beneficial compared to those only targeting one product. More information is needed to understand the specificities of use in each population to create tailored interventions.

A suicidal attempt was not contributing to the gap in daily smoking between high school students and apprenticeship. Although daily smoking was very high among those who attempted suicide, the small number of self-reported suicidal attempts could explain those results. In the literature, poor mental health during childhood is negatively correlated with educational achievement [43] and smoking is associated with depression and anxiety with no clear causal direction [44]. Using measures of mental health other than suicidal attempts could bring more insights on the relationship between education, mental health and smoking.

The unexplained part in the decomposition analyses includes the effect of unmeasured characteristics as well as the effect of the apprenticeship setting on daily smoking at 17. The latter effect could include different parameters such as leaving the classic educational system, being more in contact with adults, having a work experience at a young age, etc. Cigarette may be used as a coping mean in a challenging and stressful professional environment [45] and it may also be endorsed as the result of a process of socialization through imitation [46]. Because the unexplained part was nearly always higher than 50%, the apprenticeship setting could be one of the main reasons why apprentices smoke more than high-school students. Although more research is needed to identify potential unmeasured characteristics impacting daily smoking at 17, we are confident our results support targeted interventions among apprentices. Different smoking prevention interventions in vocational schools have been designed notably in Denmark, such as a settings-based health promotion approach through everyday school practices and school tobacco policies [47, 48] and Australia via electronic feedback and referral to online and telephone services [49]. In France, the TABADO program [50] aims at encouraging apprentices to engage in a smoking cessation program. At the 12-month follow-up, smokers enrolled in the program were more likely to have become abstinent compared to the controls (odds-ratio: 1.8; 95CI = 1.05–3.0). The P2P program [51], also targeting vocational students, for which an evaluation is ongoing, is using a peer-to-peer approach and relies on the theory of planned behaviour.

In this paper we used a Oaxaca-Blinder approach to study the determinants of the disparities between apprentices and high school students in regards to daily smoking. It is important to highlight that there are different techniques that could be used to decompose the disparity between 2 groups including mediation analysis [52–54]. Yet, it has been shown that OBD and mediation analyses provide similar conclusions with similar causal assumptions [55].

The causal pathways between education and smoking in youth are still unclear: educational disparities in smoking are present before education is completed [56]. Smoking behaviours are even likely to have been initiated before entering a general or a vocational curriculum as suggested by a Norwegian study where smoking was found to be more prevalent among youths planning vocational studies [11]. In our data, although we do not know for sure if apprentices were already smoking before starting apprenticeship, their self-reported age at smoking initiation and daily smoking is on average lower than 16 (average age for starting apprenticeship). In that respect, tobacco control programs targeting younger populations could also be effective in reducing educational disparities in smoking in adolescents. Students attending high schools or apprentice training centers are captive and easier to reach, so these two environments are conducive to interventions targeting educational disparities in smoking.

### Strength and limitations

Our analysis is based on a unique dataset representative of all French adolescents aged 17, while most surveys interviewing adolescents tend to be school-based, thus missing adolescents out of the general educational system. With an original approach using a decomposition method, we aim to fill the gap in the literature about educational disparities in smoking among adolescents. That being said, some limitations have to be acknowledged. First, in addition to accounting for the effect of the apprenticeship setting, the unexplained part could also include the effect of some unmeasured characteristics (such as perceived peer smoking, perceived accessibility of cigarettes or specific youth socialization). Second, since our data are cross-sectional our analyses cannot conclude on causal relationships between the measured covariates and the gap in daily smoking. Third, although some variables may be determinant of the educational track (e.g. parental smoking, parental occupational class), others may be determined by the educational track (e.g. money received, other drug use). Fourth, the discrepancies in predictors for the 2017 a and b samples do not allow for comparing the effects of parental smoking and amount of money received in 2017. Asking different questions to different samples of adolescents allows for multiplying the topics covered in the survey without extending the length of the questionnaire but it does come with disadvantages. Finally, French high schools are divided in general and vocational high schools. Among French high school students, smoking differences also exist depending on their type of high schools [12]. This information was not available in ESCAPAD, and more research is needed to investigate smoking disparities within high school students.

## Conclusions

Large educational disparities in daily smoking at 17 are reported between French apprentices and high school students. We highlighted which characteristics may explain them, such as other drug use, sum of money received, parental smoking and the apprenticeship setting. Such disparities are likely to persist or worsen into adulthood thus preventive measures among adolescents, especially those targeting apprentices, could help reduce educational disparities in smoking and thus disparities in health among adults.

## Supplementary information

**Additional file 1: Table S1.** Oaxaca-Blinder decomposition of differences in daily smoking between high school students and apprentices in the 2008 sample without the variables “parental smoking”: estimates and percentage contributions. **Table S2.** Oaxaca-Blinder decomposition of differences in daily smoking between high school students and apprentices in 2008 without the variable “money received”: estimates and percentage contributions. **Table S3.** Oaxaca-Blinder decomposition of differences in daily smoking between high school students and apprentices in the 2008, 2017-A and 2017-B samples. Sensitivity analysis with one model for each independent variable: estimates and percentage contributions.

## Data Availability

The data that support the findings of this study are available from OFDT (French Monitoring Centre for Drugs and Drug Addiction) but restrictions apply to the availability of these data, which were used under agreement with OFDT for the current study, and so are not publicly available. Data are however available from the authors upon reasonable request and with permission of OFDT.

## Abbreviations

JDC: National Defence and Citizenship Day
CNIS: National Council for Statistical Information
SMD: Standardized Mean Differences
