# Supplementary material for: Why do apprentices smoke much more than high school students? Understanding educational disparities in smoking with a Oaxaca-blinder decomposition analysis
Source: BMC Public Health. 2020 Jun 12;20:924. doi: 10.1186/s12889-020-09050-4 (PMC7291761; doi:10.1186/s12889-020-09050-4)
Supplement: Supplementary file 1 — Additional file 1: Table S1. Oaxaca-Blinder decomposition of differences in daily smoking between high school students and apprentices in the 2008 sample without the variables “parental smoking”: estimates and percentage contributions. Table S2. Oaxaca-Blinder decomposition of differences in daily smoking between high school students and apprentices in 2008 without the variable “money received”: estimates and percentage contributions. Table S3. Oaxaca-Blinder decomposition of differences in daily smoking between high school students and apprentices in the 2008, 2017-A and 2017-B samples. Sensitivity analysis with one model for each independent variable: estimates and percentage contributions. [file 12889_2020_9050_MOESM1_ESM.pdf]

Why do apprentices smoke much more than high school students? Understanding educational disparities in smoking with a Oaxaca-blinder decomposition analysis.

Supplementary materials

Table 1S - Oaxaca-Blinder decomposition of differences in daily smoking between high school students and apprentices in the 2008 sample without the variables “parental smoking”: estimates and percentage contributions. ESCAPAD survey, OFDT

	Model including all characteristics			Model excluding use of alcohol and cannabis		
	Estimate	Share	CI	Estimate	Share	CI
	Log odds	(%)		Log odds	(%)	
Total Unexplained	-0.14	59.5	58.4 to 61.3	-0.16	67.7	64.9 to 72.4
Total Explained	-0.10	40.5	38.7 to 41.6	-0.08	32.3	27.6 to 35.1
Age	0.00	1.7	0.6 to 2.3	-0.01	2.2	1.3 to 2.8
Gender (Girl)	0.01	-4.9	-10.2 to -1.4	0.00	1.6	-2.4 to 4.0
Parental occupational status						
Low	0.00	0.4	-0.5 to 0.9	0.00	0.0	0.0 to 0.6
Disadvantaged	0.00	0.0	-2.0 to 1.3	0.00	-0.1	-2.4 to 1.2
Intermediate	0.00	0.1	-0.3 to 0.4	0.00	0.3	-0.2 to 0.6
Advantaged	0.00	0.4	-1.1 to 1.4	0.00	-0.3	-2.1 to 0.8
High	0.00	-0.2	-2.6 to 1.4	0.00	-0.9	-3.8 to 0.8
Parents living together (Yes)	0.00	1.6	0.5 to 2.3	-0.01	3.4	2.6 to 3.9
Living alone (Yes)	0.00	0.4	-0.1 to 0.7	0.00	0.7	0.0 to 1.1
Suicide attempt (Yes)	0.00	1.5	0.6 to 2.1	-0.01	2.5	1.6 to 3.1
Grade repetition (Yes)	-0.01	5.9	1.8 to 8.6	-0.01	3.8	-1.4 to 6.9
Cannabis in the past month (Yes)	-0.04	17.4	17.0 to 18.1			
Alcohol in the past month (Yes)	-0.01	4.1	3.4 to 4.5			
Sum of money received (continuous)	-0.03	11.9	3.9 to 17.1	-0.05	19.1	12.0 to 23.4

CI: confidence interval

Table 2S - Oaxaca-Blinder decomposition of differences in daily smoking between high school students and apprentices in 2008 without the variable “money received”: estimates and percentage contributions. ESCAPAD survey, OFDT

	Model including all characteristics			Model excluding use of alcohol and cannabis		
	Estimate	Share	CI	Estimate	Share	CI
	Log odds	(%)		Log odds	(%)	
Total Unexplained	-0.16	66.7	65.2 to 68.7	-0.20	79.9	76.8 to 84.2
Total Explained	-0.08	33.3	31.3 to 34.8	-0.05	20.1	15.8 to 23.2
Age	0.00	1.9	0.8 to 2.6	-0.01	2.7	1.6 to 3.4
Gender (Girl)	0.01	-2.2	-6.2 to 0.8	-0.01	4.0	1.0 to 6.2
Parental occupational status						
Low	0.00	0.2	-0.6 to 0.8	0.00	-0.1	-1.0 to 0.6
Disadvantaged	0.00	-0.6	-2.7 to 1.0	0.00	-1.1	-3.4 to 0.6
Middle	0.00	0.1	-0.3 to 0.3	0.00	0.3	-0.2 to 0.6
Advantaged	0.00	0.3	-1.2 to 1.4	0.00	-0.2	-1.8 to 1.0
High	0.00	-0.4	-2.7 to 1.3	0.00	-1.6	-4.2 to 0.4
Parents living together (Yes)	0.00	0.4	-1.0 to 1.4	0.00	1.8	0.6 to 2.7
Living alone (Yes)	0.00	0.2	-0.2 to 0.6	0.00	0.4	-0.3 to 0.8
Suicide attempt (Yes)	0.00	1.7	0.7 to 2.5	-0.01	2.6	1.4 to 3.4
Grade repetition (Yes)	-0.01	3.6	-1.3 to 7.1	0.00	0.9	-4.6 to 4.9
Cannabis in the past month (Yes)	-0.04	16.0	15.1 to 16.6			
Alcohol in the past month (Yes)	-0.01	4.0	3.1 to 4.7			
Father smokes (Yes)	-0.01	3.6	2.3 to 4.6	-0.01	4.1	2.9 to 5.0
Mother smokes (Yes)	-0.01	4.5	3.2 to 5.4	-0.02	6.3	5.2 to 7.1

CI: confidence interval

Table 3S – Oaxaca-Blinder decomposition of differences in daily smoking between high school students and apprentices in the 2008, 2017-A and 2017-B samples. Sensitivity analysis with one model for each independent variable: estimates and percentage contributions. ESCAPAD survey, OFDT

	2008 sample			2017-A sample			2017-B sample		
	Estimate	Share	CI	Estimate	Share	p-value	Estimate	Share	CI
	Log odds	(%)		Log odds	(%)		Log odds	(%)	
Age	-0.01	3.0	2.0 to 3.9	0.00	-0.4	-1.5 to 0.5	0.00	0.3	-0.7 to 1.0
Gender (Girl)	0.00	2.0	-1.1 to 4.5	-0.02	10.0	2.4 to 14.5	0.00	1.8	-7 to 7.4
Parental occupational status									
Low	0.00	0.5	-0.2 to 1.2	0.00	-0.8	-3.2 to 1.0	0.00	-1.1	-3.4 to 0.6
Disadvantaged	0.00	-0.8	-2.1 to 0.5	0.01	-2.8	-8.4 to 1.0	0.00	-1.2	-4.9 to 1.6
Intermediate	0.00	0.4	0.0 to 0.7	0.00	0.0	-1.0 to 0.6	0.00	-0.1	-0.7 to 0.3
Advantaged	0.00	-0.4	-1.5 to 0.7	0.00	-1.1	-6.5 to 2.5	0.01	-2.7	-8.2 to 1.2
High	0.00	-0.7	-2.2 to 0.9	0.01	-2.7	-15.9 to 4.9	0.00	-1.3	-7.7 to 3.1
Parents living together (Yes)	-0.01	4.7	3.6 to 5.7	-0.01	6.0	2.9 to 8.1	-0.01	3.8	1.0 to 5.8
Living alone (Yes)	0.00	0.7	-0.1 to 1.3	0.00	0.7	-0.6 to 1.7	0.00	1.3	-0.7 to 2.8
Suicide attempt (Yes)	-0.01	2.3	1.2 to 3.2	0.00	0.1	-0.5 to 0.5	0.00	1.5	-0.1 to 2.6
Grade repetition (Yes)	-0.02	6.9	3.0 to 10	0.00	0.9	-9.3 to 7.1	0.00	0.1	-9.9 to 6.3
Cannabis in the past month (Yes)	-0.05	20.6	19.3 to 21.7	-0.05	23.5	20.7 to 25.4	-0.04	18.0	15.0 to 20.1
Alcohol in the past month (Yes)	-0.02	7.3	6.0 to 8.4	-0.02	10.8	7.6 to 12.9	-0.04	14.3	11.4 to 16.3
Father smokes (Yes)	-0.02	6.8	5.6 to 7.9				-0.02	8.5	4.8 to 10.9
Mother smokes (Yes)	-0.02	8.5	7.2 to 9.6				-0.03	11.5	8.0 to 13.8
Sum of money received (continuous)	-0.06	22.9	17.7 to 26.4	-0.04	20.3	0.3 to 27.6			

CI: confidence interval
